# Autosomal Recessive Cutis Laxa 1C Mutations Disrupt the Structure and Interactions of Latent TGFβ Binding Protein-4

**DOI:** 10.3389/fgene.2021.706662

**Published:** 2021-09-03

**Authors:** Yasmene F. Alanazi, Michael P. Lockhart-Cairns, Stuart A. Cain, Thomas A. Jowitt, Anthony S. Weiss, Clair Baldock

**Affiliations:** ^1^Wellcome Trust Centre for Cell Matrix Research, Division of Cell Matrix Biology and Regenerative Medicine, School of Biological Science, Faculty of Biology, Medicine and Health, Manchester Academic Health Science Centre, University of Manchester, Manchester, United Kingdom; ^2^Charles Perkins Centre, University of Sydney, Darlington, NSW, Australia; ^3^School of Life and Environmental Sciences, Darlington, NSW, Australia; ^4^Sydney Nano Institute, The University of Sydney, Darlington, NSW, Australia

**Keywords:** latent TGFβ binding protein-4, fibrillin, fibulin-4, tropoelastin, small angle X-ray scattering

## Abstract

Latent TGFβ binding protein-4 (LTBP4) is a multi-domain glycoprotein, essential for regulating the extracellular bioavailability of TGFβ and assembly of elastic fibre proteins, fibrillin-1 and tropoelastin. LTBP4 mutations are linked to autosomal recessive cutis laxa type 1C (ARCL1C), a rare congenital disease characterised by high mortality and severely disrupted connective tissues. Despite the importance of LTBP4, the structure and molecular consequences of disease mutations are unknown. Therefore, we analysed the structural and functional consequences of three ARCL1C causing point mutations which effect highly conserved cysteine residues. Our structural and biophysical data show that the LTBP4 N- and C-terminal regions are monomeric in solution and adopt extended conformations with the mutations resulting in subtle changes to their conformation. Similar to LTBP1, the N-terminal region is relatively inflexible, whereas the C-terminal region is flexible. Interaction studies show that one C-terminal mutation slightly decreases binding to fibrillin-1. We also found that the LTBP4 C-terminal region directly interacts with tropoelastin which is perturbed by both C-terminal ARCL1C mutations, whereas an N-terminal mutation increased binding to fibulin-4 but did not affect the interaction with heparan sulphate. Our results suggest that LTBP4 mutations contribute to ARCL1C by disrupting the structure and interactions of LTBP4 which are essential for elastogenesis in a range of mammalian connective tissues.

## Introduction

Latent TGFβ binding protein-4 (LTBP4) is a large secreted multi-domain glycoprotein that is associated with fibrillin, the major component of extracellular matrix (ECM) microfibrils, in the ECM. Two major isoforms of LTBP4 are expressed in mammalian cells, a long (LTBP4L) and short form (LTBP4S; Saharinen et al., 1998), produced by alternative splicing (Kantola et al., 2010). Both isoforms share similar domain structure, but LTBP4L has an additional amino-terminal 4-cysteine domain. The long and short LTBP4 isoforms have overlapping expression in the lung and aorta, whereas, in the skin and heart, LTBP4S is the major expressed isoform (Bultmann-Mellin et al., 2015). Moreover, LTBP4S has higher expression in lung during late embryonic and postnatal periods (Bultmann-Mellin et al., 2017).

Mutations in both LTBP4 isoforms lead to autosomal recessive cutis laxa type 1C (ARCL1C), initially named Urban-Rifkin-Davis Syndrome, and an ARCL1C-like phenotype is seen in LTBP4 deficient mice (Sterner-Kock et al., 2002; Urban et al., 2009; Bultmann-Mellin et al., 2015). ARCL1C patients share similar clinical and pathological features of craniofacial, pulmonary, gastrointestinal and genitourinary anomalies accompanied with generalised cutis laxa. Both ARCL1C patients and LTBP4 deficient mice have high postnatal mortality due to respiratory failure caused by severely disrupted pulmonary elastic fibre architecture (Urban et al., 2009; Bultmann-Mellin et al., 2015), indicating a crucial role for LTBP4 in elastic fibre assembly. LTBP4 regulates extracellular TGFβ bioavailability by covalent interaction with the small latent TGFβ complex (Saharinen and Keski-Oja, 2000; Annes et al., 2004).

**Figure 1:**
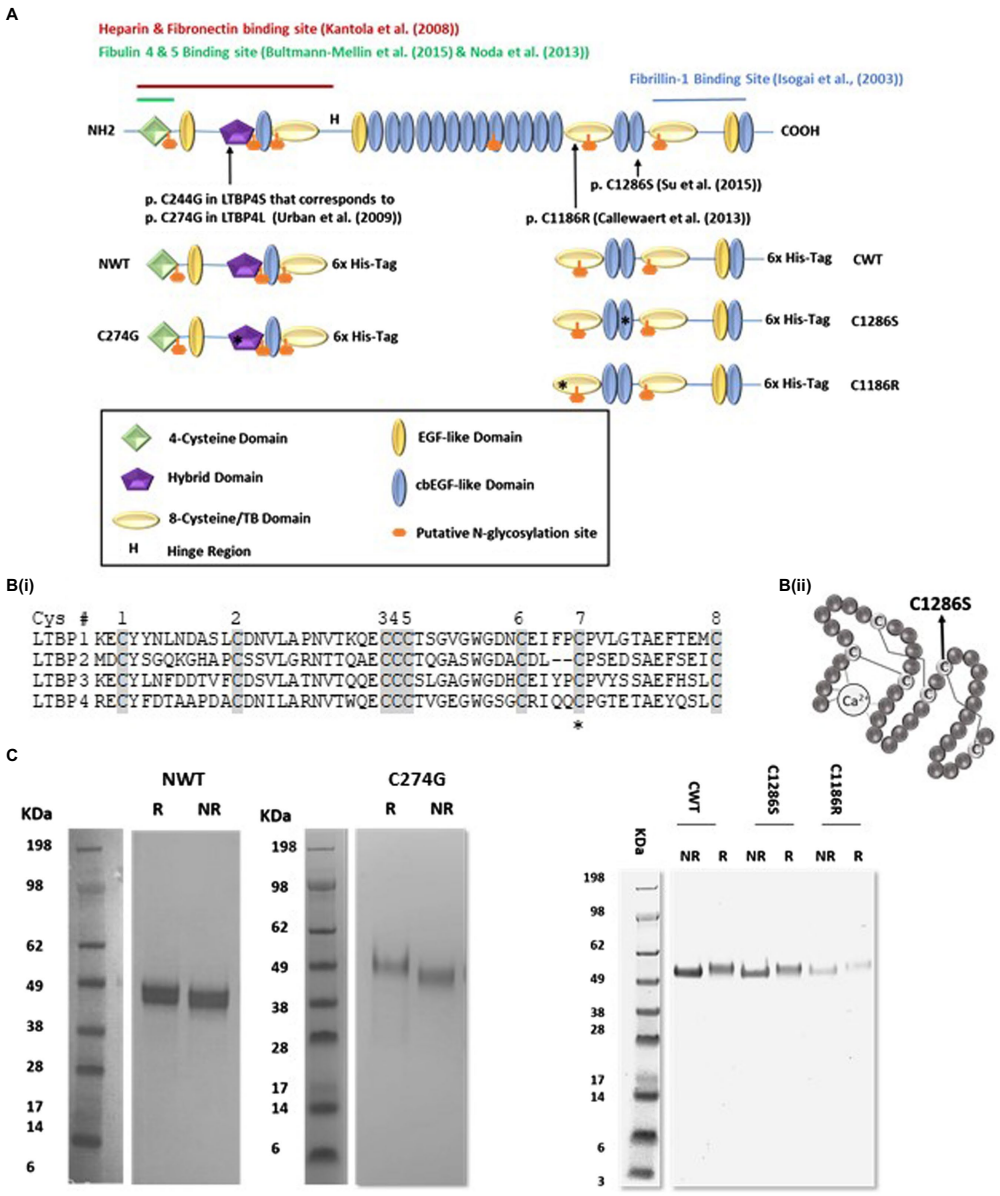
Expression and purification of recombinant latent TGFβ binding protein-4 (LTBP4). **(A)** Schematic diagram of the domain organisation of full-length human LTBP4S with interaction sites indicated and the LTBP4 constructs with 6x-His-tag used in this study. Arrows and asterisks indicate the positions of the ARCL1C mutations. **(B)** (i) Amino acid sequence of the second TB domain in LTBP1-4 with the eight conserved cysteine residues shaded. C1186 is the C7 residue (highlighted with an asterisk), involved in an intramolecular disulphide bond. C2 and C6 are involved in the interaction with TGFβ-LAP. (ii) Schematic diagram of a representative cbEGF domain with conserved cysteine residues labelled and disulphide bonds illustrated by lines. The position of the substituted cysteine, C1286, C5 is indicated by an arrow. **(C)** SDS-PAGE of the purified LTBP4 N- and C-terminal constructs: NWT, C274G, CWT, C1286S and C1186R under reducing (R) and non-reducing (NR) conditions.

However, LTBP4 is primarily involved in the regulation of elastic fibre assembly in a TGFβ independent manner (Dabovic et al., 2009). LTBP4 modulates elastic fibre assembly by interacting with other extracellular partners, such as fibulin-4 (Bultmann-Mellin et al., 2015, 2016), fibulin-5 (Noda et al., 2013) and fibrillin-1 (Ono et al., 2009). The LTBP4 C-terminal region binds directly to the N-terminal region of fibrillin-1 (Isogai et al., 2003; Ono et al., 2009) and this interaction is essential for deposition of LTBP4 in the matrix (Ono et al., 2009; Zilberberg et al., 2012). The LTBP4 domain structure (Figure 1A), as for other LTBP family members (LTBP1, 2 and 3), is highly structurally related to fibrillin. Both LTBPs and fibrillin share two types of cysteine-rich domains, the six-cysteine epidermal growth factor-like domains (EGF) that form disulphide bonds in a conserved 1–3, 2–4 and 5–6 pattern and eight-cysteine TGFβ binding protein-like (TB) domains that form disulphide bonds in a conserved 1–3, 2–6, 4–7 and 5–8 pattern (Yuan et al., 1997; Lack et al., 2003). The LTBPs also share a 4-cysteine domain near their N-terminus. LTBP4 has 20 EGF-like domains, of which 17 are calcium binding (cbEGF), four TB domains and a proline-rich interdomain region.

Here, we focused on three ARCL1C missense mutations that affect highly conserved cysteine residues, p.C1186R (Callewaert et al., 2013) and p.C1286S (Su et al., 2015) in the C-terminal region within the second TB and the 16th cbEGF domains, respectively, and p.C274G in the N-terminal hybrid domain. A cutis laxa (CL) patient with homozygous LTBP4 p.C1186R mutations suffered a life-threatening pulmonary defect hydronephrosis with generalised skin laxity (Callewaert et al., 2013). The p.C1286S mutation was reported in a 14-year-old female heterozygous for p.G793Efs*5 (resulting in a premature termination codon) and p.C1286S with the clinical and pathological characteristics of CL. Skin biopsies show large globular elastin aggregates and poor integration into the fibrillin microfibrils (Su et al., 2015). Whereas the p.C1186R mutation resulted in severe ARCL1C, the C1286S mutation resulted in mild to moderate pulmonary disease (Su et al., 2015). The homozygous point mutation p.C274G was first reported in a 4-month male with generalised LTBP4-related CL (Urban et al., 2009) and severe pulmonary emphysema caused by severely disrupted elastic fibres (Ritelli et al., 2019). Our hypothesis was that mutations in LTBP4 may result in structural changes and altered molecular interactions of LTBP4 to result in defective elastic fibre assembly.

To determine whether ARCL1C mutations interfere with LTBP4 structure and molecular interactions, we analysed the structure of the LTBP4 N- and C-terminal regions using complementary structural and biophysical methods and compared its structural features with the ARCL1C mutants. We identified tropoelastin as a new interaction partner for LTBP4 and investigated the impact of ARCL1C mutations on the interactions with heparan sulphate, fibulin-4, tropoelastin and fibrillin-1 to further understand the role of LTBP4 in elastic fibre assembly and disruption in ARCL1C.

## Materials and Methods

### Protein Expression and Purification

Human wild-type and mutant LTBP4S N- and C-terminal constructs were purchased as gene strings (residues 29–394 and 1,114–1,557) and ligated into a modified version of the mammalian expression vector pCEP4 (Invitrogen), pCEP-Pu/AC7 that contains a C-terminal six-histidine residue tag (6x His-tag). The recombinant human wild-type and mutant LTBP4 constructs were stably transfected and expressed in Human Embryonic Kidney (293)-EBNA cells. The proteins were then purified using nickel affinity column (GE Healthcare) followed by size-exclusion chromatography on an AKTA purifier FPLC using a Superdex 200 10/300GL column (GE Healthcare) equilibrated with buffer containing 10 mm Tris, 150 mm NaCl and pH 7.4 at a flow rate of 0.5 ml/min. Protein identity was confirmed by mass spectrometry (MS) and western immunoblotting and concentration determined using BCA protein assay (Thermo Fisher Scientific). Purified proteins were digested using PNGase-F according to the manufacturer’s protocol (New England’s Biolabs). The removal of glycans was verified using SDS-PAGE. Full-length fibulin-5, fibulin-4, fibronectin and tropoelastin (SHELdelta26A) were expressed and purified as previously described (Martin et al., 1995; Choudhury et al., 2009; Cheng et al., 2018). Tropoelastin (SHELdelta26A) corresponds to amino acid residues 27–724 of GenBank entry AAC98394 isoform (Vrhovski et al., 1997).

### Multi-Angle Light Scattering

Purified protein samples (0.5 ml at approximately 0.4–1 mg/ml) were loaded into a Superdex 200 10/300GL column (GE Healthcare) running at a flow rate of 0.5 ml/min using a buffer containing 10 mm Tris, 150 mm NaCl and pH 7.4. Eluted protein fractions were passed from the column into a Wyatt DAWN Heleos II EOS 18 angle laser photometer with QELS detector (Wyatt Technologies) connected to an Optilab T-rEX refractive index detector. The absolute molar mass, concentration and hydrodynamic radius of the resulting peaks were analysed using ASTRA 6.

### Sedimentation Velocity Analytical Ultracentrifugation

Monomeric protein samples (0.4–1 mg/ml) in the same buffer as used in multi-angle light scattering (MALS) were characterised by sedimentation velocity AUC using a Beckman XL-A analytical ultracentrifuge with an An60Ti 4-hole rotor running at 45,000 rpm at 20°C. The sedimenting boundary was monitored at either 280 or 230 nm for 200 scans. Data were analysed by continuous model-based distribution C(s) of Lamm equation solutions method using SEDFIT software (Schuck, 2000), and the resulting sedimentation coefficients were corrected to standard conditions using SEDNTERP software (Philo, 1997).

### Circular Dichroism

The purified wild-type and mutant LTBP4 samples, at a concentration range of 0.5–1 mg/ml in 10 mm Tris, 150 mm NaCl and pH7.4 buffer, were analysed by CD using the far-UV spectral region (190–260 nm). The far-UV spectra were recorded in millidegrees (mdeg) by a Jasco-J810 spectropolarimeter. Measurements were taken every 0.2 mm in a 0.5 cm path length cell at 25°C. Ten accumulated scans were recorded, averaged and corrected for each sample. The secondary structure content was quantitatively analysed by the online tool Dichroweb^1^ using CDSSTR and CONTIN algorithms.

### Small Angle Light Scattering

In-line SEC-SAXS was performed on purified proteins at a concentration range of 1–2 mg/ml in the same buffer used for MALS and sedimentation velocity analytical ultracentrifugation (SV-AUC). CWT and C1286S small angle X-ray scattering (SAXS) data were collected at 1 s intervals using 45ul purified protein sample passed through a Superdex 200 3.2/300 column at beamline BM29, European synchrotron radiation facility (Grenoble, France), while SAXS data for NWT, C274G and C1186R were collected at B21 at Diamond. SAXS data were pre-processed and reduced using in-house software at each beamline. For each frame with a consistently similar radius of gyration (Rg) across the elution peak, the protein scattering intensities were merged. SAXS data were then analysed and buffer scattering subtracted from that of the sample using ScAtter software.^2^ The Rg of the protein was estimated by Guinier analysis and the intraparticle distance distribution function P(r) in real space was evaluated using the Indirect Fourier transform (IFT) program GNOM, and particle shapes were modelled *ab initio* using DAMMIN software in slow mode (Svergun, 1999). For each construct, 10 *ab initio* models were generated to check for reproducibility and the models were within two standard deviations of mean of the normalised spatial discrepancy.

### Surface Plasmon Resonance

The binding between the fibrillin-1 PF3 fragment (residues 1–722; Rock et al., 2004) and the LTBP4 C-terminal region was analysed in real time using the surface plasmon resonance (SPR) ProteOn system at 25°C. Fibrillin-1 was diluted in 10 mm HEPES, 150 mm NaCl and pH 7.4 supplemented with 0.005% (v/v) Tween-20 and then injected at serial dilutions from 100–0 nm over a constant concentration of immobilised LTBP4 C-terminal region on amine coupling GLC sensor chip using the manufacturer’s instructions (Bio-Rad). All binding experiments for each LTBP4 construct were performed at least two times. The sensor chip surface was regenerated using 10 mm glycine at pH 2.5. Equilibrium analysis was used to determine kinetic parameters.

### Biolayer Interferometry

Binding studies were performed on an OctetRED96 system (ForteBio) using biolayer interferometry (BLI). Streptavidin biosensors were hydrated in 10 mm HEPES, 150 NaCl and pH 7.4, 0.005% (v/v) Tween-20 for 10 min then loaded with biotinylated ligand at constant concentration. The loaded biosensors were incubated with serial dilutions of analyte in the same buffer used for hydration. The biosensors were regenerated using 10 mm glycine at pH 2.5. The background response was subtracted from all binding sensorgrams and all experiments performed in solid black 96 well plates at 25^°^C with an agitation speed of 1,000 rpm and repeated at least twice. The binding kinetics were analysed using Octet software version 7 (ForteBio). The goodness of binding data fitting was assessed by *χ*^2^ and *R*^2^ values. Biotinylated human long chain heparan sulphate was kindly provided by the Biomolecular analysis facility in the University of Manchester.

## Results

### Expression and Purification of the Human LTBP4 N- and C-terminal Regions

To structurally characterise the LTBP4 N- and C-terminal regions, HEK293-EBNA cells stably expressing the wild-type LTBP4 N- (NWT) and C-terminal regions (CWT) and ARCL1C mutants (C274G, C1186R and C1286S) were generated (Figures 1A,B). The LTBP4 proteins were purified using nickel affinity followed by size-exclusion chromatography (SEC; Supplementary Figure S1) with similar elution volumes for CWT and mutants and NWT and C274G mutant. To determine whether the loss of a cysteine residue resulted in aberrant dimerisation for the mutants, samples were analysed using non-reducing and reducing SDS-PAGE. Only monomeric species were observed for both the wild type and mutants with the same shift upon reduction indicating that the free cysteine residue in the mutants does not result in dimerisation or gross perturbation of folding (Figure 1C). For the N-terminal samples, the main species appeared slightly larger than the sequence predicted size (NWT 40 kDa), whereas for the C-terminal samples, they were closer to their sequence predicted size (CWT 57 kDa). Full-length LTBP4S has six putative N-glycosylation sites based on sequence but NetNGlyc server prediction suggests only four of these are utilised (Gupta and Brunak, 2002), with the C-terminal construct predicted to contain one N-linked glycan and the N-terminal construct three N-linked glycans. The presence of N-linked sugars was verified experimentally using PNGase-F treatment which resulted in a lower molecular weight for all LTBP4 constructs, but with a larger shift in size apparent for the N-terminal constructs (Supplementary Figure S2) suggesting more glycosylation in the N-terminal region consistent with the prediction.

### The C-terminal Region of LTBP4 Is Elongated and Monomeric

**Figure 2:**
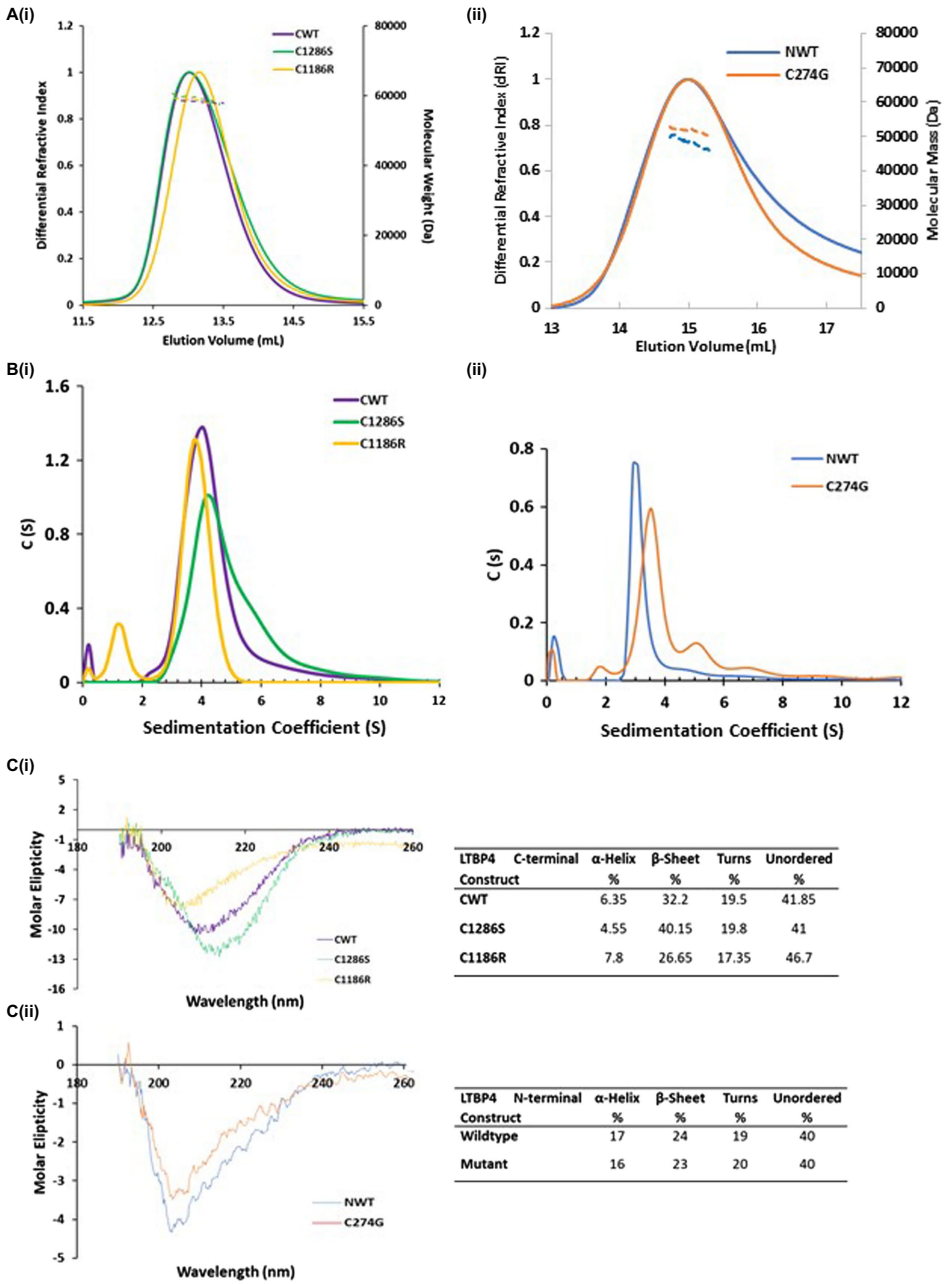
Hydrodynamic properties and secondary structural characterisation of LTBP4. **(A)** SEC-MALS chromatograms in which the differential refractive index (solid lines) and molecular weight (dashed lines) are plotted as a function of elution volume (ml). The colours are purple (CWT), gold (C1186R), green (C1286S), blue (NWT) and orange (C274G) and this colour scheme is used in all figures. All constructs are monomeric in solution with mass of 58 kDa for the LTBP4 C-terminal constructs (i), and 47 and 51 kDa for the NWT and C274G constructs (ii), respectively. **(B)** Sedimentation velocity profiles from AUC for the (i) C-terminal and (ii) N-terminal LTBP4 constructs. **(C)** The far-UV-CD spectra in the 260–190 nm range for the wild type and mutant LTBP4 (i) C-terminal and (ii) N-terminal constructs. CD data show that the spectra have a negative maxima characteristic of proteins with a high β-sheet content and unordered conformation. The C1286S substitution had little impact on the CD spectra, whereas a more pronounced difference was observed for C1186R. Ten scans were recorded for each sample. The table summarises the secondary structure content of the wild-type and mutant constructs.

Our recent study on human LTBP1 demonstrated that the N-terminal region oligomerises into dimers and higher-ordered species in a calcium-dependent manner (Troilo et al., 2016). MALS in combination with SEC and SV-AUC was used to determine the size, oligomeric state and hydrodynamic properties of the LTBP4 constructs and ARCL1C causing mutants. SEC-MALS data showed a single peak for all constructs with mass of 47 kDa and 51 kDa for NWT and C274G, respectively, and mass of 58 kDa for CWT, C1186R and C1286S indicating that they are all monomeric in solution (Figure 2A). The hydrodynamic radius was 4.7 nm for both CWT and C1286S and 5.0 nm for C1186R (Table 1).

**Table 1:** Experimental hydrodynamic results for LTBP4 C-terminal constructs.

Hydrodynamic properties	NWT	C274G	CWT	C1286S	C1186R
Molecular mass (kDa)^a^	47	51	58	58	58
Hydrodynamic radius R_h_ (nm)^a^	ND	ND	4.7	4.7	5.0
Sedimentation coefficient (Sw)^b^	3.15	3.54	4.12	4.24	3.82
Sedimentation coefficient (Sw,20)^b^	3.25	3.61	4.05	4.16	3.75
Frictional ratio^b^	1.40	1.33	1.39	1.31	1.49
R_h_ (nm)^b^	3.20	3.14	3.70	3.61	3.83
Radius of gyration R_g_ (nm)^c^	3.6	3.5	3.9	4.0	4.2
Maximum dimension D_max_ (nm)^c^	18.0	15.7	18.0	18.0	18.7

a Multi-Angle Light Scattering (MALS).

b Sedimentation Velocity Analytical Ultracentrifugation (SV-AUC).

c Small-Angle X-ray Scattering (SAXS).

The AUC data confirmed that constructs were monomeric, but the AUC profile for C1286S had a small shoulder and the profile for C274G small amounts of higher sedimenting species indicating an increased tendency to form higher-ordered species (Figure 2B). CWT sediments at 4.05 Svedberg (*S*_20W_) and NWT sediments at 3.25 *S*_20W_ both with frictional ratios of 1.4 higher than that expected for a globular protein suggesting a moderate elongation of these regions. However, the C274G mutant sedimented faster than the wild type counterpart (3.61 *S*_20W_) with lower frictional ratios (1.33) indicative of a more compact structure. In contrast, the C-terminal mutants had similar hydrodynamic properties to the wild-type protein.

### ARCL1C Mutation Alters the Secondary Structure of the LTBP4 C-terminal Region

To gain more information on the structure of the LTBP4 C-terminal region, we performed circular dichroism (CD). Secondary structure was analysed using far UV-CD which showed a negative maximum at 205 nm for NWT and 209 nm for CWT, characteristic of a high content of β-sheet and unordered regions (Figure 2C). The C1286S substitution had little impact on the far UV-CD spectra with a shift of 3 nm in the position of the negative maxima, whereas a change was observed for C1186R, resulting in a shift of 6 nm in the position of the negative peak, suggestive of a more unordered structure (Figure 2Ci). Using the online tool Dichroweb, the secondary structure content was quantitatively analysed and summarised. The estimated secondary content for both NWT and C274G was similar. For CWT, the estimated α-helical content was lower (6.35%), whereas there was a higher proportion of β-sheet (32.2%) and unordered regions (61.4%), consistent with the expected secondary structure of cbEGF/TB domains. The C1186R mutant had a decrease in β-sheet content to 26.6% and an increase in unordered structure to 64%. These data indicate that the C1186R mutation alters the secondary structure of the LTBP4 C-terminal region, possibly resulting in a less stable protein.

### The LTBP4 C-terminal Region Has an Elongated and Flexible Nanostructure

**Figure 3:**
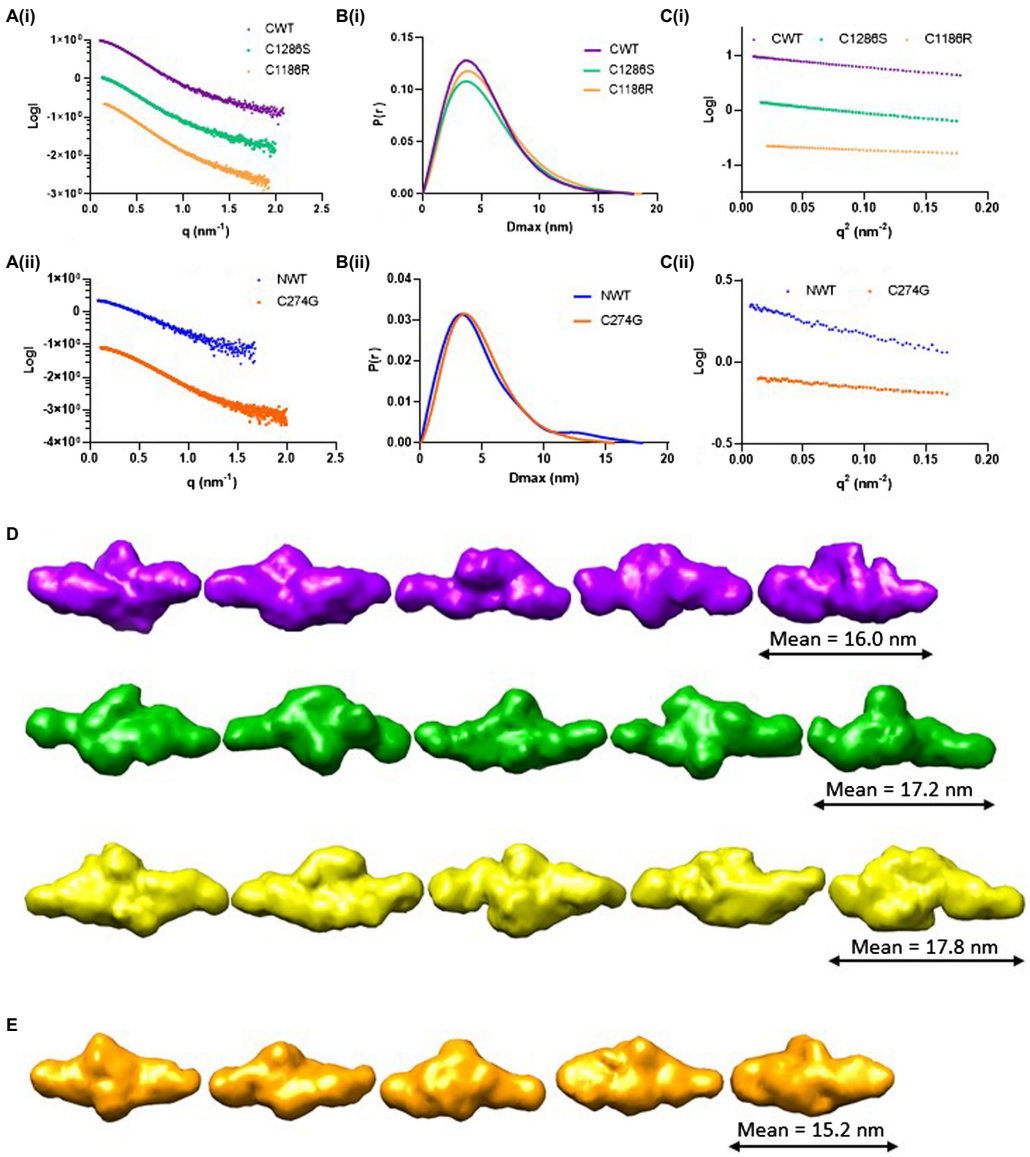
Structural characterisation of LTBP4 N- and C-terminal regions using small angle X-ray scattering. **(A)** The experimental 1D scattering intensity I plotted as a function of q for (i) CWT, C1286S and C1186R, and (ii) NWT and C274G constructs used for analysis and modelling. **(B)** The IFT of I vs. q using GNOM, P(r) vs. r for wild type and mutants indicate the maximum dimensions in real space. **(C)** Guinier plot of the low q region for the (i) CWT, C1286S and C1186R and (ii) NWT and C274G showing linearity. **(D)** Five example *ab initio* models generated by DAMMIN are shown each for CWT (purple), C1286S (green) and C1186R (gold) and **(E)** C274G (orange). Models are shown to the same scale and the average end-to-end length is indicated for each construct.

To obtain more detailed structural information, SAXS was performed. SAXS data were collected directly from SEC eluates to ensure the monodispersity of LTBP4 in solution. The 1D scattering intensity data of each construct are plotted as a function of q (Figure 3A). DATCMP was used to compare the wild-type scattering data to each of the mutants which calculated *p* values below 2.5 e-83 indicating that the data are statistically significantly different (Franke et al., 2015). Guinier plots were used for sample quality assessment and determination of the radius of gyration (Rg) which showed linearity in the Guinier region indicating that no aggregation was present (Figure 3C). The Rg was similar for CWT and C1286S (3.9 nm and 4.0 nm, respectively), whereas the Rg for C1186R was slightly larger (4.2 nm).

The flexibility of the LTBP4 C-terminal region was assessed and the normalised Kratky plots show a single peak, with maxima slightly deviating from the expected position for a globular protein, indicating that this region has an elongated structure with some flexibility rather than a compact, globular conformation (Supplementary Figure S3). The distance distribution function P(r), shown in Figure 3B, is also consistent with an extended conformation for the LTBP4 C-terminal region. The maximum dimension was 18.0 nm, 18.0 nm and 18.7 nm for CWT, C1286S and C1186R, respectively. A*b initio* bead models of the wild type and mutants were generated using DAMMIN (Figure 3D). The *ab initio* models are consistent with an elongated conformation but with differences between CWT and mutant constructs. The average end-to-end distance of the WT models was 16.0 nm, whereas the mutants were more extended (C1186R average end-to-end distance = 17.8 nm and C1286S average end-to-end distance = 17.2 nm). The structural parameters obtained from the experimental data are summarised in Table 1.

For NWT and C274G, the *R*_g_ was 3.6 and 3.5 nm, respectively. The obtained distance distribution functions P(r) shown in Figure 3B, also suggests an extended conformation with maximum dimension of 18 nm for the NWT and 15.7 nm for the C274G mutant. The scattering intensity at higher angles was insufficient for NWT to perform further data analysis so the data for C274G were analysed where a*b initio* models confirmed the elongated structure (Figure 3E). The flexibility of the N-terminal region was assessed, the Porod-Debye plot plateaued at high q range, indicating that this region is not flexible (Supplementary Figure S3). The normalised Kratky plot displayed single peak with maxima slightly deviating from globularity point, indicating that this region is asymmetric and elongated (Supplementary Figure S3).

### ARCL1C Mutation Reduces Binding to Fibrillin-1

**Figure 4:**
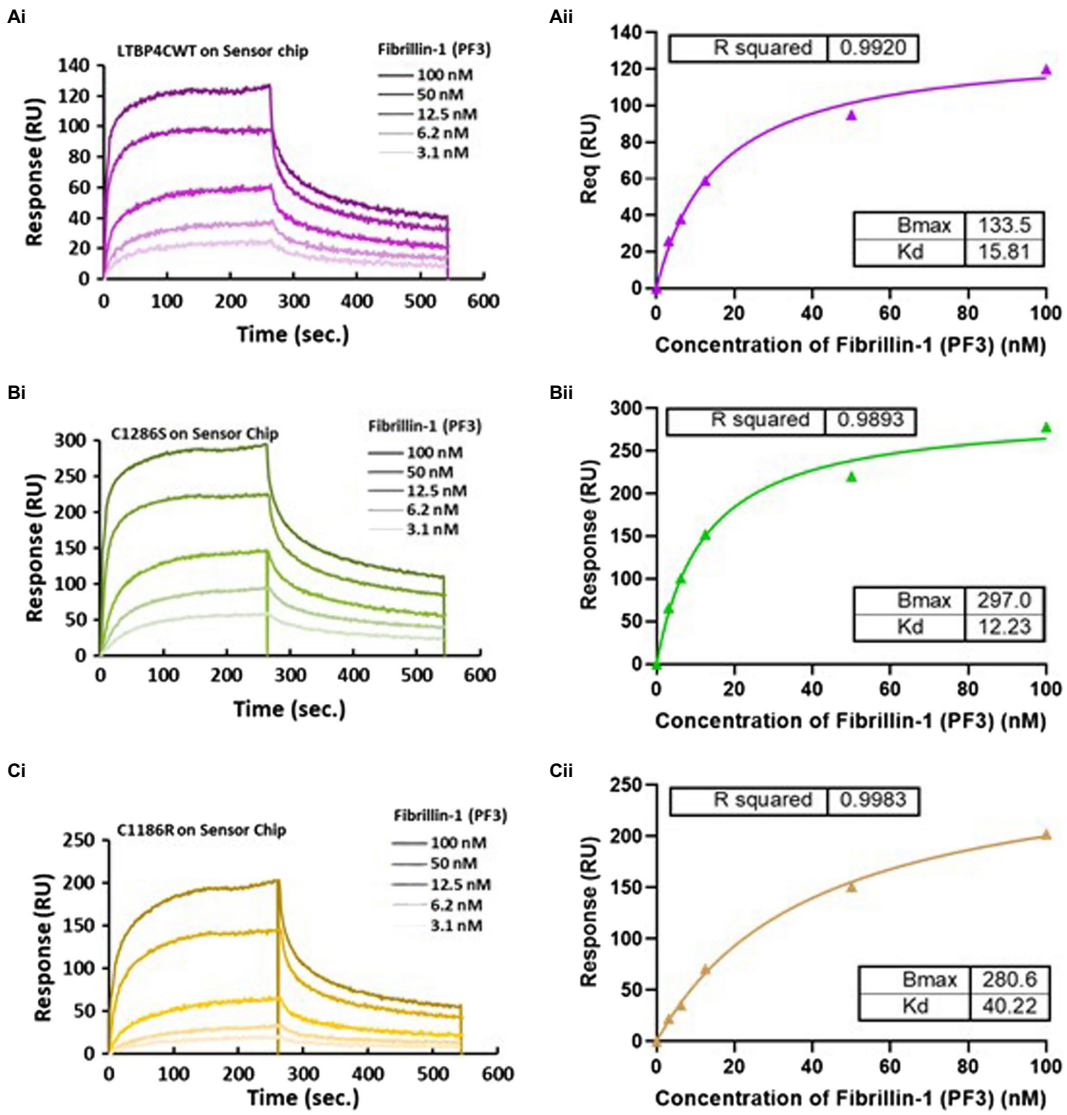
Surface plasmon resonance analysis of the fibrillin-1 PF3 construct binding to LTBP4. **(A)** CWT, **(B)** C1286S, and **(C)** C1186R. LTBP4 was immobilised on the sensor chip (GLC) using amine coupling and purified fibrillin-1 (PF3) was injected as analyte at different concentrations (100–3.1 nm) over the immobilised LTBP4. The binding affinity was determined by steady state with equilibrium response plotted against fibrillin-1 concentration as shown in panel (ii) for all constructs. All experiments were performed in at least duplicate and representative curves shown.

LTBP4 binds to fibrillin-1 as has been previously shown by solid and solution phase binding studies (Isogai et al., 2003; Ono et al., 2009). These studies demonstrated that binding was *via* the TB3, EGF3 and cbEGF17 domains in LTBP4 (domains indicated in Figure 1A). The LTBP4-fibrillin-1 interaction is essential for matrix assembly and incorporation of LTBP4 (Ono et al., 2009; Zilberberg et al., 2012) so ARCL1C causing mutations could interfere with this important interaction. Therefore, to investigate the impact of ARCL1C mutations on the interaction with fibrillin-1, SPR binding analysis was performed. An N-terminal region of fibrillin-1 [PF3 fragment as previously described (Rock et al., 2004)] encompassing the LTBP4 binding site was used. Fibrillin-1 was injected at different concentrations over immobilised LTBP4 C-terminal constructs. The kinetic analysis demonstrated that fibrillin-1 strongly interacted with both CWT and mutants with low nanomolar affinity (*K_D_*). The calculated binding affinities for fibrillin-1 to C WT and C1286S were equivalent (15.81 and 12.23 nm, respectively), indicating that C1286S substitution did not affect this interaction (Figure 4), whereas the C1186R mutant had a slightly weaker binding affinity with a *K_D_* of 40.22 nm, indicating that C1186R substitution slightly interfered with the fibrillin-1 interaction. Moreover, these data suggest that the second TB domain in LTBP4 where residue C1186 is located might be involved in this interaction or that the conformational change induced by the mutation may have longer-range effects.

### The C-terminal Region of LTBP4 Directly Interacts With Tropoelastin Which Is Perturbed by ARCL1C Mutation

**Figure 5:**
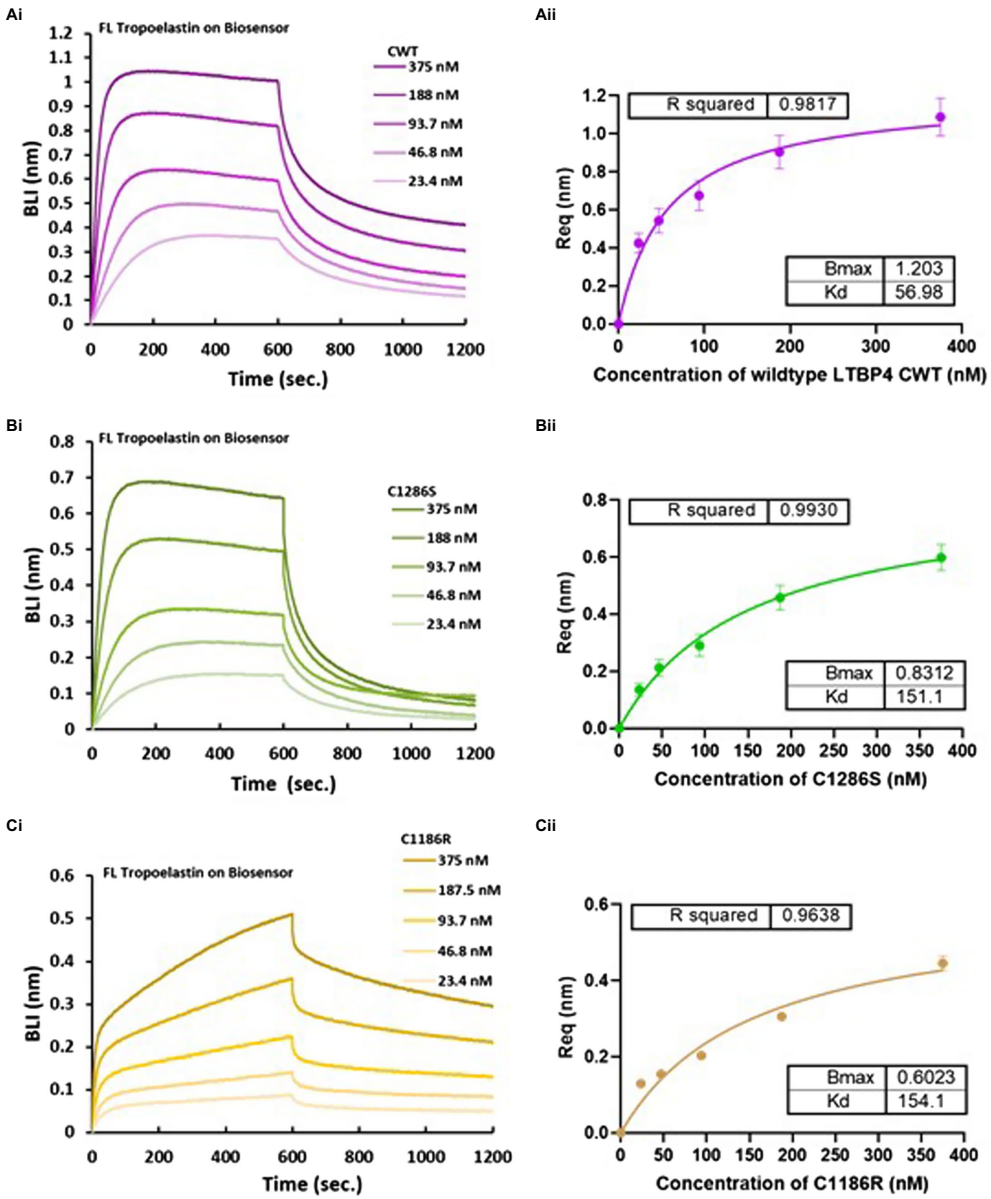
Biolayer interferometry (BLI) analysis of LTBP4CT binding to full-length tropoelastin. OctetRED analysis shows that LTBP4 **(A)** CWT, **(B)** C1286S and **(C)** C1186R at different concentrations (375–23.4 nm) directly bind to immobilised full-length tropoelastin. The binding affinity was determined by steady state analysis (equilibrium) plotted against **(A)** (ii) CWT, **(B)** (ii) C1286S and **(C)** (ii) C1186R concentration. All experiments were performed in at least duplicate and averaged *K_D_* in nm are presented. Error bars represent standard deviation.

A previous study showed that LTBP4 knockdown prevented elastin deposition in human dermal fibroblasts and LTBP4 has also been shown to interact with tropoelastin indirectly *via* fibulin-5 (Noda et al., 2013). We wanted to test whether LTBP4 might interact directly with tropoelastin. Binding studies using OctetRED96 BLI were performed using N- and C-terminal constructs of LTBP4 to determine if either region binds to tropoelastin. The sensorgrams for the N-terminal region showed no binding response to immobilised tropoelastin (data not shown), whereas the C-terminal region bound to tropoelastin. The kinetics of the interaction between CWT and full-length tropoelastin were studied by incubating immobilised tropoelastin with increasing concentrations of CWT (Figure 5). The binding analysis demonstrated that CWT binds to tropoelastin with binding affinity of 57 nm. The effect of ARCL1C mutations on this interaction was then investigated. Both mutants C1286S and C1186R showed weaker binding affinity to tropoelastin with a *K_D_* of 151.1 and 154.1 nm, respectively. Although the steady state analysis for both mutants resulted in a similar dissociation constant for binding to tropoelastin, the kinetic analysis demonstrated a highly biphasic association and slower dissociation steps for C1186R binding to tropoelastin (Figure 5). These data indicate that the C1186R mutation interferes with tropoelastin binding more than the C1286S mutation. We further confirmed the interaction between the C-terminal region of LTBP4 and tropoelastin by performing binding studies in the opposite orientation, immobilising the LTBP4 C-terminal constructs and using tropoelastin as the analyte (Supplementary Figure S4).

### The Effect of ARCL1C Causing Point Mutation on Binding to Fibulin-4

**Figure 6:**
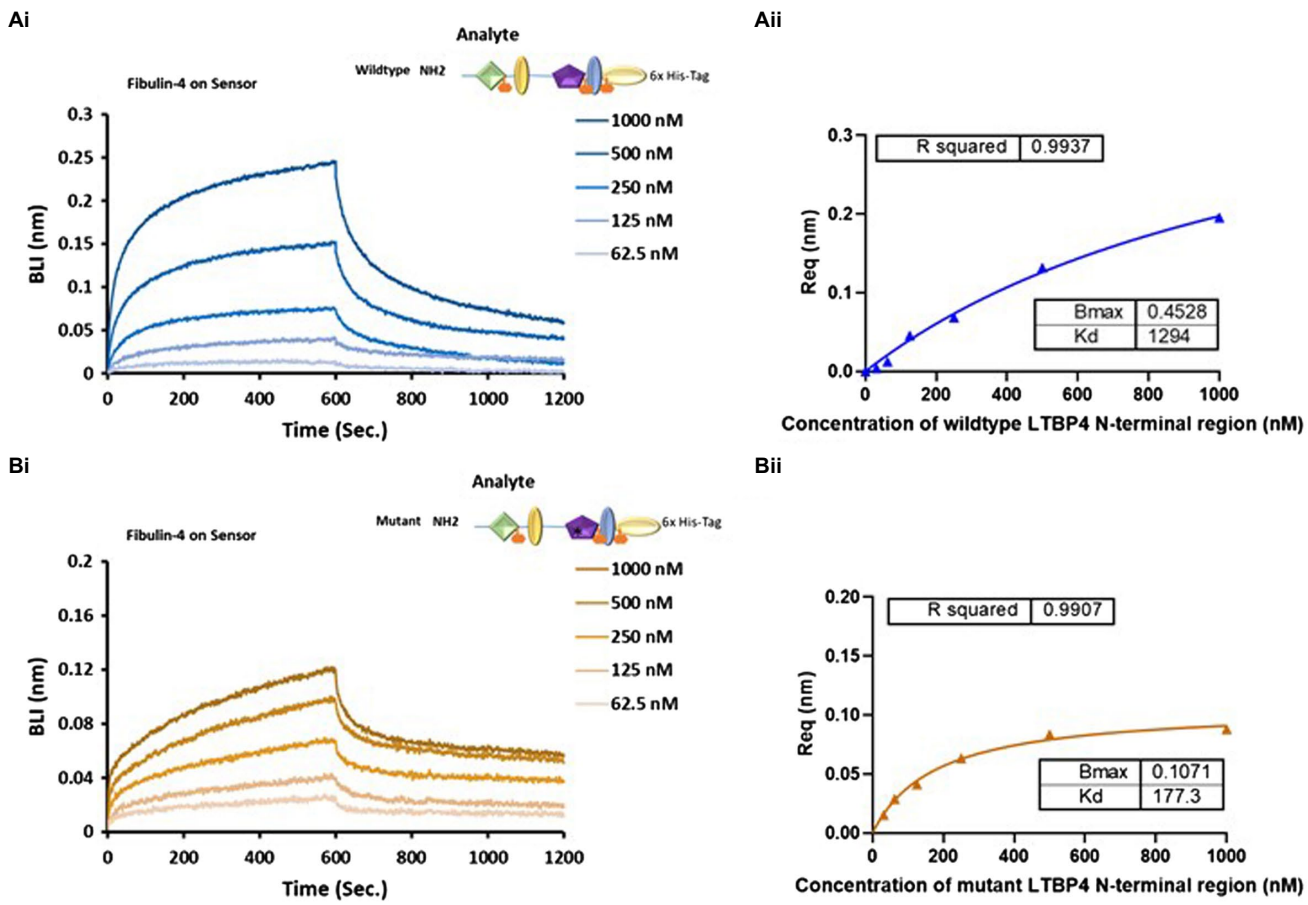
The impact of ARCL1C causing point mutation on LTBP4 N-terminal interaction with fibulin-4. Binding studies using BLI were performed using OctetRED analysis. Representative Octet sensorgrams showing both **(A)** (i) NWT and **(B)** (i) C274G at a range of concentrations (1000–62.5 nm) bind to immobilised fibulin-4. The binding affinity *KD* was determined by steady state (equilibrium) for **(A)** (ii) the wild type and **(B)** (ii) C274G. All experiments were performed in at least duplicate and representative results are shown.

LTBP4 interacts with fibulin-4 to support elastogenesis where both LTBP4 long and short isoforms bind to fibulin-4 *via* the N-terminal region (Bultmann-Mellin et al., 2015). To test whether the interaction between the N-terminal region and fibulin-4 is affected by the ARCL1C mutation, we performed kinetic analysis using BLI. As shown in Figure 6, both NWT and C274G bound to immobilised full-length fibulin-4. Interestingly, C274G bound to fibulin-4 with a *K_D_* of 177.3 nm compared to a *K_D_* of 1,294 nm for NWT which is a 7-fold increase in binding affinity. NWT had faster association and dissociation steps compared to C274G (Figure 6A) suggesting that this mutation makes the fibulin-4 binding site more accessible.

### The N-terminal Region Does Not Bind to Fibronectin but Interacts With Heparan Sulphate

Fibronectin directs the assembly and deposition of many ECM proteins. LTBP4 deposition is thought to be mediated *via* an interaction between its N-terminal region and fibronectin. An interaction between the LTBP4 N-terminal region and plasma fibronectin has previously been observed using solid phase binding assays and deletion of the N-terminal region prevented interaction with fibronectin (Kantola et al., 2008). To our knowledge, no previous studies have examined this interaction using real-time binding methods. To determine whether NWT was able to bind fibronectin, we performed binding studies using BLI. However, no binding was detected between immobilised NWT and full-length fibronectin (data not shown).

**Figure 7:**
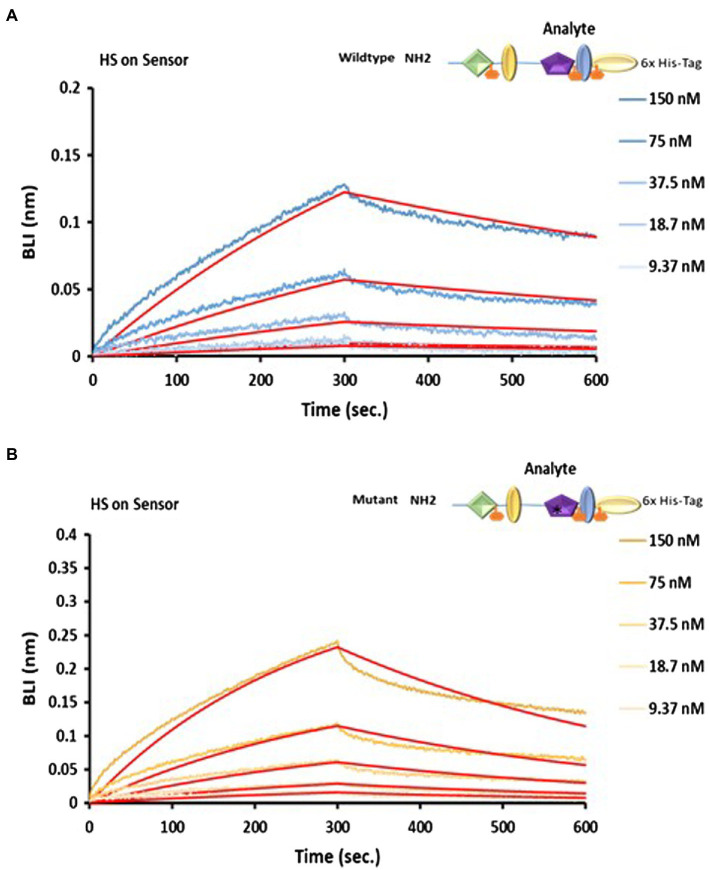
Analysis of LTBP4 N-terminal interaction with fibronectin and HS using BLI. OctetRED sensorgrams showing both **(A)** the wild type and **(B)** mutant N-terminal region at a range of concentrations (150–9.37 nm) bind to immobilised heparin. The binding affinity *KD* was 52.6 nm for the wild type and 53.4 nm for the mutant, determined by fitting to a 1:1 Langmuir binding model (red curves). All experiments were performed in at least duplicate and representative results are shown.

Heparan sulphate (HS) has been reported to mediate binding between LTBP1 and fibronectin (Chen et al., 2007) as no direct interaction occurs between LTBP1 and fibronectin. Therefore, we hypothesised that HS might also mediate the interaction between LTBP4 and fibronectin. The importance of heparin/heparan sulphate (HS) interaction in mediating cell adhesion has been previously studied (Bax et al., 2007) and using heparin affinity chromatography, an interaction between the LTBP4 N-terminal region and heparin has previously been demonstrated (Kantola et al., 2008). To determine the kinetics of a HS-LTBP4 interaction and whether the ARCL1C mutation interferes with this interaction, we examined binding using BLI. HS was immobilised then incubated with NWT and the sensorgrams show that NWT interacts with HS with high affinity (*K_D_* = 53.4 nm; Figure 7A). The mutant LTBP4 N-terminal region also binds to HS with similar affinity *K_D_* = 52.6 nm (Figure 7B).

## Discussion

LTBP4 is important for the correct formation of an intact elastic fibre network with mutations resulting in ARCL1C, confirming its biological significance in elastogenesis (Urban et al., 2009). In our study, we have provided structural and functional information on the LTBP4 N- and C-terminal regions and the effect of ARCL1C mutations. Both N- and C-terminal regions were monomeric in solution but the N-terminal region had a tendency to form higher-ordered species. This is consistent with our previous study on LTBP1, where the LTBP1 terminal regions were also monomeric but the LTBP1 N-terminal region readily multimerised in the presence of calcium (Troilo et al., 2016). Biophysical data show that the N- and C-terminal regions were elongated; however, the incorporation of a C274G mutation in the hybrid domain induced a more compact conformation. This finding is consistent with a previous study on substitutions occurring in the hybrid domain of fibrillin-1 that resulted in the production of more compact proteins compared with the wild type (Mellody et al., 2006).

Small angle X-ray scattering data showed that the N-terminal region had an elongated inflexible conformation. This is consistent with our recent study on the N-terminal region of LTBP4 homolog, LTBP1, which also adopts an elongated and inflexible conformation (Troilo et al., 2016). The C-terminal region was also elongated with some flexibility. This finding is consistent with our recent study on the C-terminal region of LTBP1 which also adopts an elongated and flexible conformation (Troilo et al., 2016). The secondary structure was consistent with TB and cbEGF domain structures, which are mainly composed of β-sheets and small amount of α-helix (Rao et al., 1995). The C1286S mutation resulted in a slight increase in the β-sheet content, while the C1186R mutant resulted in a lower β-sheet and higher-unordered structure content. Together, our structural data suggest that the substitution of highly conserved cysteines within the hybrid, TB or cbEGF domains, impacts the conformation of LTBP4.

The observed structural differences caused by the mutations led us to hypothesise that these mutations might also interfere with LTBP4 molecular interactions with extracellular proteins. Our SPR binding studies demonstrate that the CWT and C1286S mutant showed similar binding affinities to fibrillin-1, indicating that the substitution in domain cbEGF16 had little impact on fibrillin-1 binding. Conversely, the C1186R mutation resulted in a slight reduction in the binding to fibrillin-1, indicating that this substitution in the second TB domain might interfere with fibrillin-1 binding which could suggest that this domain contributes to the interaction with fibrillin-1 or the structural changes caused by this mutation effect binding. It has been demonstrated that fibrillin-1 is required for LTBP4 deposition (Ono et al., 2009) and so perturbing this interaction may result in altered LTBP4 deposition in the matrix.

Our wild-type LTBP4 N-terminal region bound to fibulin-4 with a *K_D_* of 1,294 nm using BLI. This interaction has previously been analysed using SPR with much higher affinity of 15.4 nm but using a much smaller construct consisting only of the first two domains of LTBP4S (the 4-cysteine and EGF domains; Bultmann-Mellin et al., 2015). It may be that the additional domains present in our constructs occlude the fibulin-4 binding site. However, the C274G construct bound to fibulin-4 with higher affinity (*K_D_* = 177.3 nm) suggesting that the conformational change induced by the mutation may result in increased accessibility of the fibulin-4 binding site. The LTBP4 interaction with fibulin-4 is vital for fibulin-4-elastin deposition into microfibril scaffolds (Bultmann-Mellin et al., 2015) and perturbation of this interaction would likely impact on elastogenesis.

Using BLI binding assays, we show that the LTBP4 C-terminal region but not the N-terminal region interacts with tropoelastin with both C1186R and C1286S mutations slightly impacting on tropoelastin binding. A previous study was not able to detect an interaction between full-length LTBP4S and tropoelastin using solid phase assays (Noda et al., 2013). However, recently full-length LTBP4L has been shown to adopt a compact conformation that becomes more open and extended after exposure to fibulin-4 (Kumra et al., 2019). Therefore, the tropoelastin binding site in full-length LTBP4 might be hidden prior to interaction with fibulin-4, whereas in our shortened LTBP4 construct, the binding site for tropoelastin might be accessible. Noda et al. also showed that fibulin-5 mediates an interaction between LTBP4 and tropoelastin (Noda et al., 2013), it is possible that fibulin-5 could also induce a conformational change in LTBP4 exposing a cryptic tropoelastin binding site. Elastic fibre formation might require both indirect and direct molecular interactions between LTBP4 and tropoelastin. This is consistent with previous studies that observed different elastic fibre ultrastructural anomalies in patients with either fibulin-5 or LTBP4 mutations. Both patients showed globular elastin deposits in skin that were poorly integrated into microfibrils, but the LTBP4 mutations resulted in larger elastin deposits and less uniform elastic fibres than fibulin-5 mutations (Callewaert et al., 2013). These findings are consistent with knockdown studies where in the absence of fibulin-5, LTBP4 was still incorporated into a fibrillar matrix (Noda et al., 2013).

LTBP4 has been suggested to interact with plasma fibronectin *via* the N-terminal region using solid phase binding methods (Kantola et al., 2008). However, we could not detect an interaction between purified cellular fibronectin and LTBP4 using BLI. Immunolocalisation studies have demonstrated that LTBP4 colocalises initially with fibronectin but then it deposits into the matrix in a fibronectin independent manner and localises with fibrillin-1 (Kantola et al., 2008). Indicating that fibronectin might be required for the initial matrix deposition of LTBP4 but not in matured tissue. Our finding is consistent with a previous study where LTBP1 interacts indirectly with fibronectin and through direct interaction with heparan sulphate (Chen et al., 2007). Using heparin affinity chromatography, LTBP4 has been shown to directly interact with heparin (Kantola et al., 2008). Our real-time binding studies confirmed a direct interaction between LTBP4 and heparan sulphate. It has been suggested that the direct interaction of LTBP4 with heparan sulphate is important for its matrix deposition (Kantola et al., 2008).

Although for the C-terminal mutations only subtle effects were observed, there was more perturbation of the C1186R mutation than the C1286S mutation. The difference in effect might be linked to the residue position and type of amino acid substitution. C1186R disrupts the C4-C7 disulphide bond involved in stabilising intramolecular folding of the second TB domain, whereas the cysteine residues involved in disulphide bonding to LAP are C2 and C6 (Lack et al., 2003). There is an important hydrophobic area between C6 and C7 responsible for binding of TGFβ-LAP (Saharinen and Keski-Oja, 2000) so the C7 substitution might disturb this area. While the C1186S mutation disrupts the C5-C6 disulphide bridge in the 16th cbEGF domain, the disulphide bridges in the cbEGFs of fibrillin-1 stabilise an antiparallel β-sheet that increases binding to calcium (Downing et al., 1996; Schrijver et al., 1999). A cysteine to serine substitution is more conservative than a cysteine to arginine substitution which introduces a positive charge. These factors could account for the differences between these mutations both in terms of structural alterations and perturbed interactions with binding partners.

In summary, we have provided the solution structure of the N- and C-terminal regions of LTBP4 and shown that ARCL1C causing point mutations in the C-terminal region result in a more elongated protein with reduced binding to tropoelastin, whereas a mutation in the N-terminal region induces a more compact conformation and increases binding to fibulin-4. Alterations to the interactions between LTBP4 and tropoelastin and fibulin-4 would likely perturb normal elastogenesis contributing to ARCL1C pathogenesis where abnormal elastin aggregates are incorrectly incorporated into fibrillin microfibril scaffolds. These data also demonstrate the importance of the conserved cysteine residues in maintaining the structure and function of the LTBP4 terminal regions.

## Data Availability Statement

The raw data supporting the conclusions of this article will be made available by the authors, without undue reservation.

## Author Contributions

CB contributed to conception and design of the study. YA performed the experimentation and analysis and wrote the first draft of the manuscript. ML-C collected and analysed the SAXS data. SC supported the protein binding experiments. TJ supported the hydrodynamic data collection and analysis. AW provided the reagents and advice on tropoelastin binding experiments. All authors contributed to manuscript revision, read and approved the submitted version.

## Conflict of Interest

AW is the founder of Elastagen which was sold to Allergan.

The remaining authors declare that the research was conducted in the absence of any commercial or financial relationships that could be construed as a potential conflict of interest.

## Publisher’s Note

All claims expressed in this article are solely those of the authors and do not necessarily represent those of their affiliated organizations, or those of the publisher, the editors and the reviewers. Any product that may be evaluated in this article, or claim that may be made by its manufacturer, is not guaranteed or endorsed by the publisher.

## Footnotes

^1^ http://dichroweb.cryst.bbk.ac.uk/html/home.shtml

^2^ http://www.bioisis.net/tutorial/9
