# Supplementary material for: Autosomal Recessive Cutis Laxa 1C Mutations Disrupt the Structure and Interactions of Latent TGFβ Binding Protein-4
Source: Front Genet. 2021 Sep 3;12:706662. doi: 10.3389/fgene.2021.706662 (PMC8446450; doi:10.3389/fgene.2021.706662)
Supplement: Supplementary file 1 [file Data_Sheet_1.PDF]

Supplementary Figures

Supplementary Figure 1

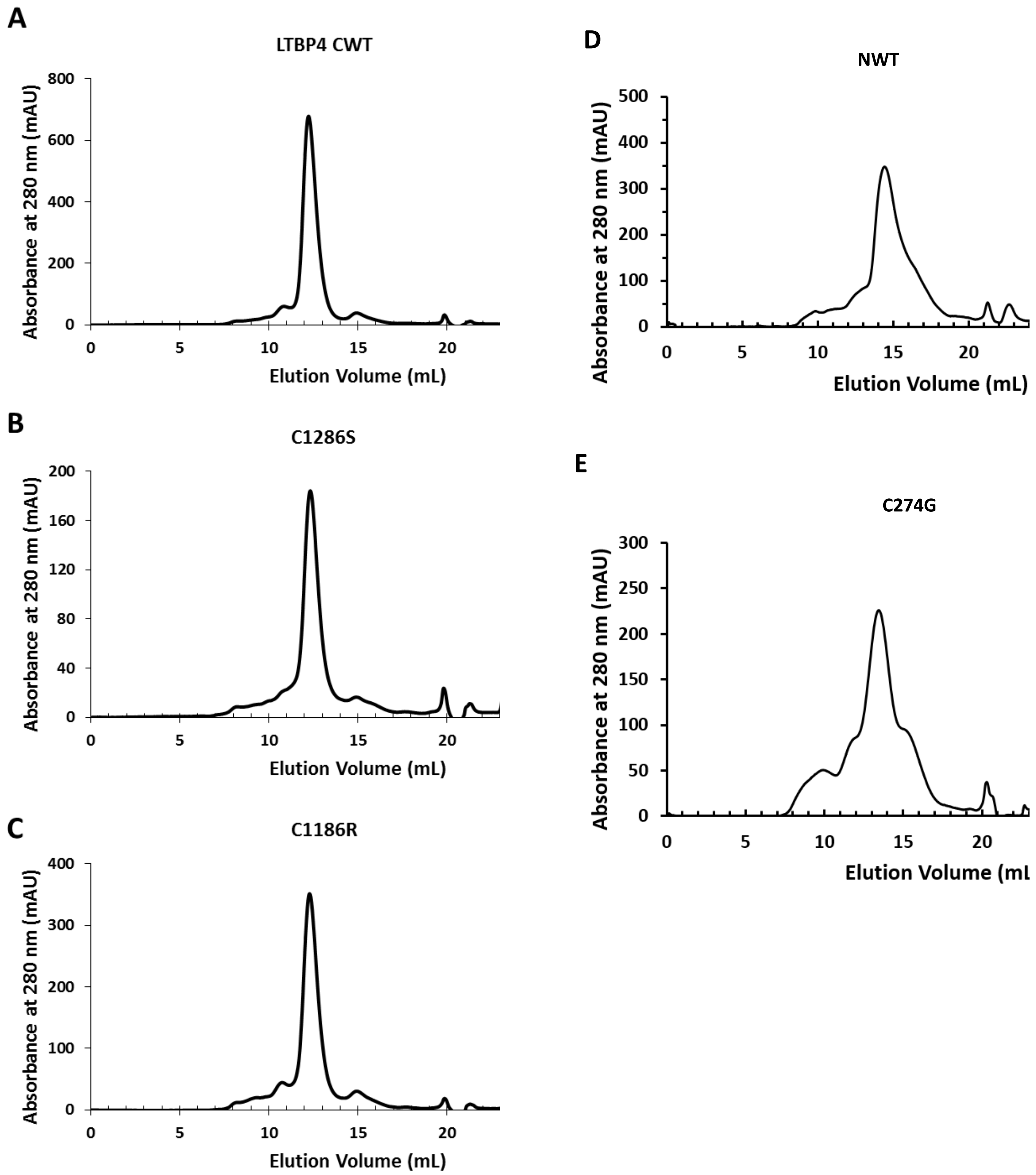

Figure 1: Size exclusion chromatograms of the **(A)** wildtype LTBP4 C-terminal (CWT), **(B)** C1286S and **(D)** C1186R showing similar elution volumes of the monomer species for each construct. **(D)** The wildtype N-terminal region (NWT) elutes later and at a similar position to the C274G construct **(E)**. All proteins were in buffer containing 10 mM Tris and 150 mM NaCl at pH 7.8 and eluted at a flow rate of 0.5 ml/min.

Supplementary Figure 2

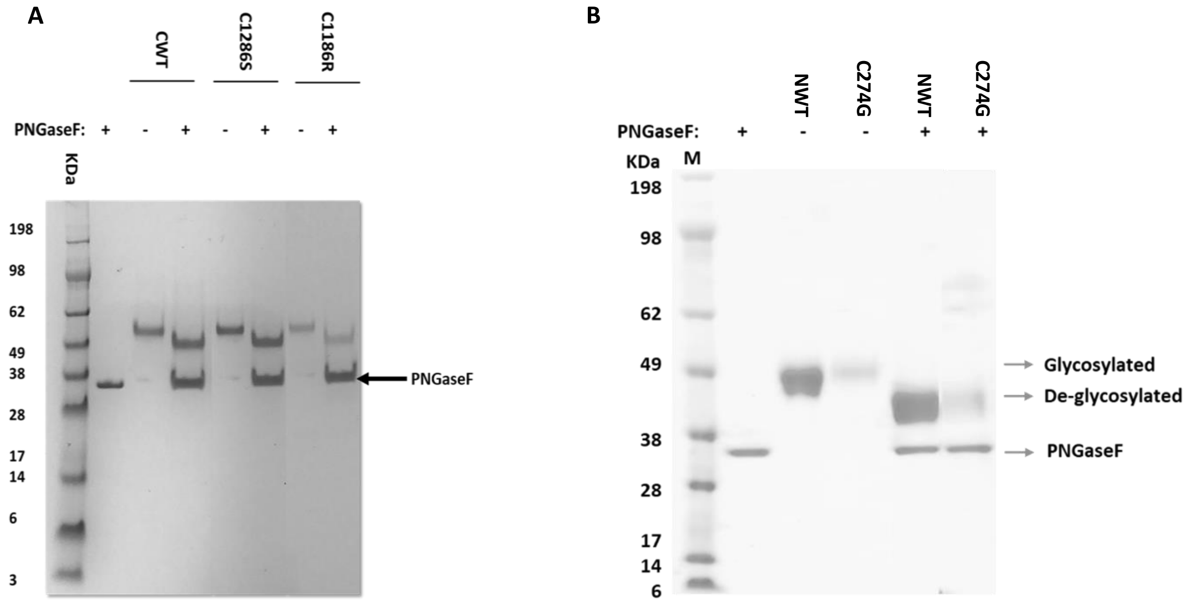

Figure 2 : LTBP4 deglycosylation with PNGaseF. Non-reduced SDS-PAGE gel showing the purified LTBP4 **(A)** C-terminal and **(B)** N-terminal constructs before and after deglycosylation with PNGaseF.

Supplementary Figure 3

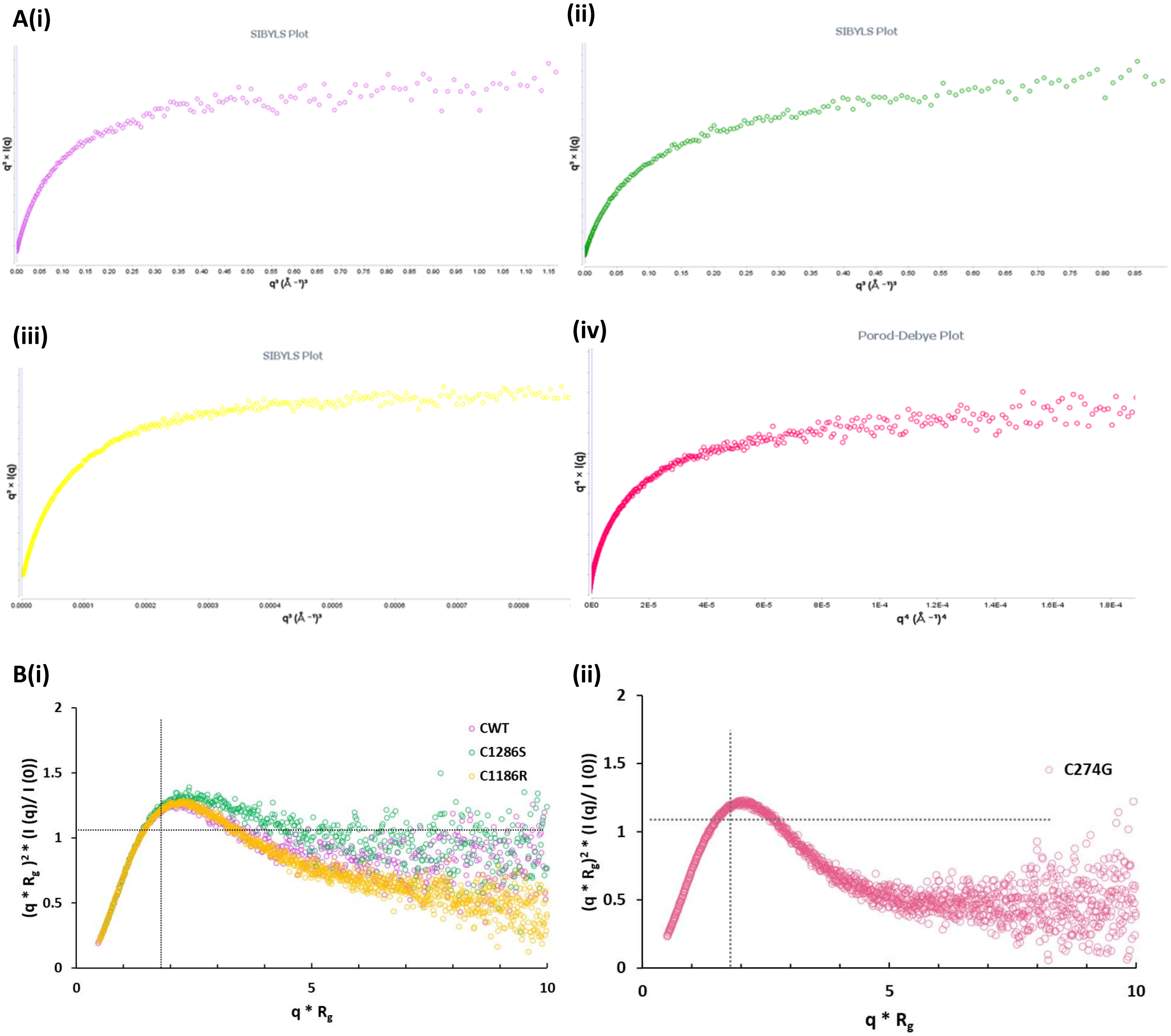

Figure 3: SAXS analysis of LTBP4 constructs. (A) Earlier plateau in the SIBYLS (q3) plot than in the Porod-Debye plot for **(i)** the wildtype LTBP4 C-terminal**, (ii)** C1286S and **(ii)** C1186R suggesting flexibility in their structures. **(iv)** Whereas the N-terminal C274G plateaus in the Porod-Debye plot (q4) indicating this region is more rigid. **(B)** The dimensionless Kratky plots for **(i)** CWT and mutants, and **(ii)** NWT and mutants show a peak deviating from the expected peak position for globular particles (cross-hair marks the Guinier-Kratky point (1.732, 1.1)), indicating that the proteins are folded and have a non-globular elongated conformation.

Supplementary Figure 4

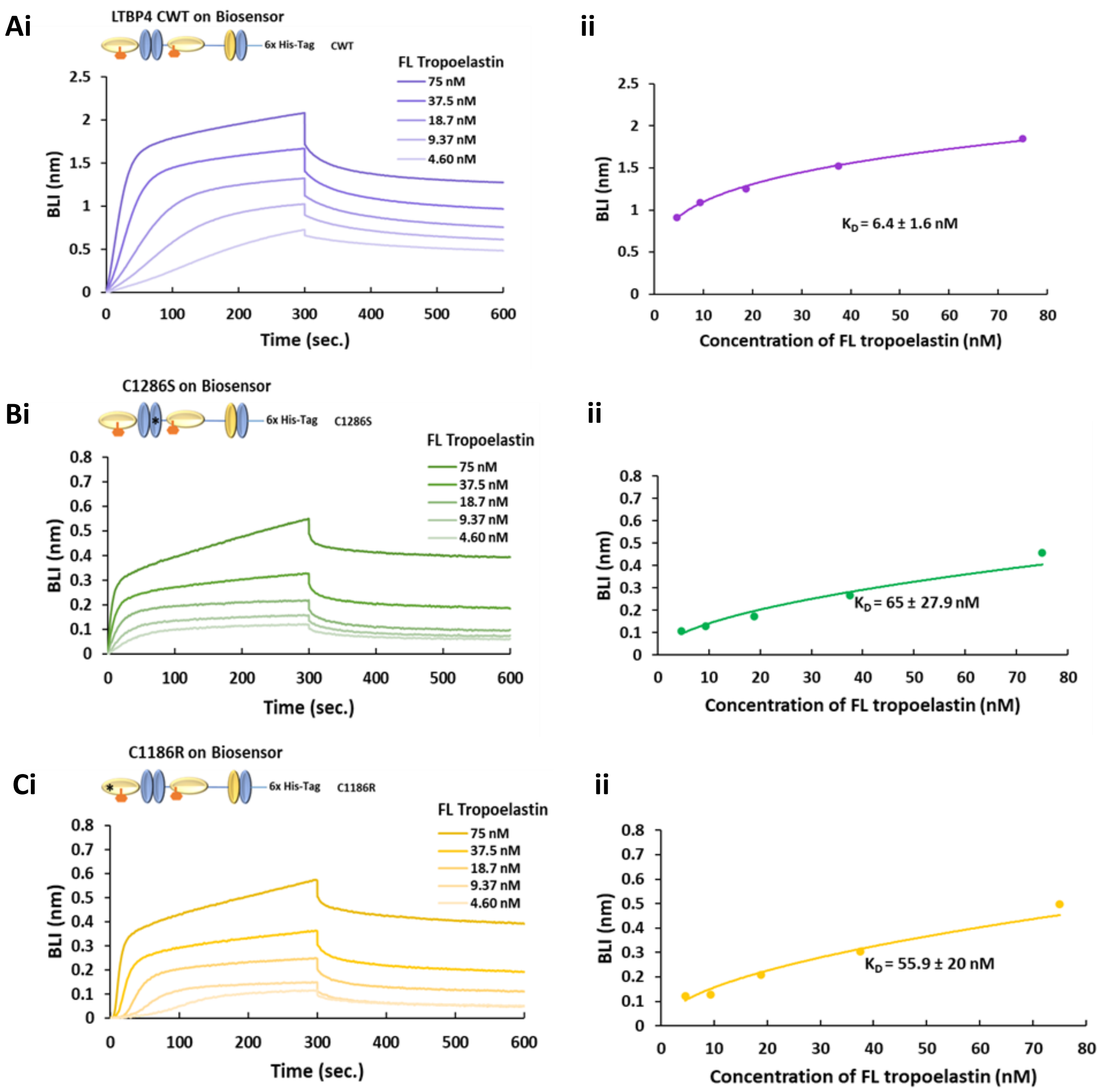

Figure 4: LTBP4 binding to full-length tropoelastin. OctetRED96 analysis shows that different concentrations (75 – 4.60 nM) of full-length tropoelastin directly bind to 100 nM of immobilized **(A)** CWT, **(B)** C1286S and **(C)** C1186R. The binding affinity was determined by steady state (equilibrium) plotted against full length tropoelastin concentration with the determined averaged binding affinity K_D for the interaction (shown in panel **(ii)** for each construct). All experiments were performed at least twice and representative results are shown.
